# Design and Methodology of a Multicenter Randomized Clinical Trial to Evaluate the Efficacy of Tongmai Jiangtang Capsules in Type 2 Diabetic Coronary Heart Disease Patients

**DOI:** 10.3389/fphar.2021.625785

**Published:** 2021-06-03

**Authors:** Yu Wang, Yilei Guo, Ye Lei, Shuwei Huang, Liping Dou, Chang Li, Buchang Zhao, Wei Fu, Peng Zhou, Haitong Wan, Mingjun Zhao, Jiehong Yang

**Affiliations:** ^1^ School of Life Sciences, Zhejiang Chinese Medical University, Hangzhou, China; ^2^ School of Basic Medical Sciences, Zhejiang Chinese Medical University, Hangzhou, China; ^3^ Department of Endocrinology and Metabolism, The Second Affiliated Hospital of Shaanxi University of Traditional Chinese Medicine, Xianyang, China; ^4^ Department of Cardiology, The First Affiliated Hospital of Zhejiang Chinese Medical University, Xianyang, China; ^5^ Department of Cardiology, The Second Affiliated Hospital of Zhejiang Chinese Medical University, Hangzhou, China; ^6^ Department of Brain and Heart CO Treatment, Xi'an Buchang Traditional Chinese Medicine Cardiac-Cerebral Diseases Hospital, Xi'an, China; ^7^ Department of Cardiac-Cerebral Diseases, Yinchuan Cardiac-Cerebral Treatment Internet Hospital, Yinchuan, China; ^8^ Institute of Brain and Heart CO Treatment, Zhejiang Chinese Medical University, Hangzhou, China; ^9^ Department of Cardiovascular, Affiliated Hospital of Shaanxi University of Traditional Chinese Medicine, Xianyang, China

**Keywords:** tongmai jiangtang capsules, Chinese medicine, type 2 diabetes mellitus, coronary heart disease, randomized controlled trial

## Abstract

**Background:** Population-based studies have consistently showed an increased incidence of coronary heart disease and cardiac mortality in patients with type 2 diabetes mellitus (T2DM). Tongmai Jiangtang capsules (TJC) are Chinese patent medicines that have been approved in China for the treatment of diabetic vascular complications. However, the evidence supporting the efficacy of Tongmai Jiangtang capsules in type 2 diabetic coronary heart disease (T2DM-CHD) remains unclear. Herein, we designed a randomized, parallel-controlled clinical trial to investigate a new complementary therapy for T2DM-CHD patients.

**Methods:** A total of 360 T2DM-CHD subjects (aged 18–75 years) will be randomly assigned to the TJC group or the placebo group at a 2:1 ratio. On the basis of western medicine therapy, all the participants will receive TJC or placebo, orally, three capsules/treatment, three per day for 12 weeks. The primary outcomes will be assessed according to the Canadian Cardiovascular Society (CCS) classification. All statistical analyses will be performed setting a two-sided 0.05 significance level, using SAS 9.4 statistical software.

**Discussion:** The efficacy of TJC for the treatment of T2DM-CHD patients will be evaluated. The study will provide reliable clinical research evidence for application of TJC in treating T2DM-CHD patients.

**Clinical Trial Registration:** https://www.chictr.org.cn/enIndex.aspx, Chinese Clinical Trial Registry ChiCTR2000037491.

## Introduction

Type 2 diabetes mellitus (T2DM) is a common non-communicable disease worldwide. According to data from the World Health Organization, the worldwide prevalence of T2DM was 2.8% in 2000 and it is expected to rise to 4.4% in 2030 (Wild et al., 2004; Guariguata, 2012). The prevalence of T2DM is also increasing rapidly in China (Zhang et al., 2018a). T2DM is associated with a high risk of cardiovascular, microvascular, and other complications (Sarwar et al., 2010). Studies have shown that T2DM is an established risk factor for coronary heart disease (CHD) (Schramm et al., 2008) and can significantly increase the risk of death (Di Angelantonio et al., 2015).

At present, studies have shown that aspirin, statins, angiotensin-converting enzyme inhibitor (ACEI), angiotensin receptor blockers (ARB), and metformin can reduce the risk of vascular complications in patients with T2DM coronary heart disease (T2DM-CHD) (Holman et al., 2008; Malahfji and Mahmarian, 2018). Despite great progresses in treatment strategies for vascular disease in T2DM-CHD, some adverse reactions and side effects may occur, such as the risk of bleeding in the elderly caused by low-dose aspirin (McNeil et al., 2018), statin-related muscle symptoms (Stroes et al., 2015), and hyperkalemia caused by ACEI and ARB, especially in T2DM patients with chronic renal insufficiency (Raebel, 2012). There is a critical need for evidence-based novel therapies.

Traditional Chinese Medicine (TCM) has a thousand-year history and an analogous history of clinical practice, TCM has accumulated rich experience and plays a significant role in treating diabetes mellitus. As a complementary therapy, TCM is promising for the treatment of vascular complications in T2DM patients and is widely used in Asian countries. Tongmai Jiangtang capsules (TJC) produced by Baoding Tianhao Pharmaceutical Co. Ltd. (Baoding, China) gained approval by the China Food and Drug Administration (CFDA) (National Medicine permission number: Z20026853) in 2002. TJC consists of Radix Pseudostellariae, Radix Salviae Miltiorrhizae, Rhizoma Coptidis, Radix Astragali, Gynostemma pentaphylla, Rhizoma Dioscoreae, Rhizoma Atractylodis, Radix Scrophulariae, Hirudo, Fructus Malvae, and Radix Puerariae. As the main components in TJC, Radix Salviae Miltiorrhizae and Radix Astragali effectively increase coronary blood flow, relieve myocardial ischemia, and inhibit platelet aggregation (Zhang 2015). A systematic review of the efficacy and safety of Radix Salviae Miltiorrhizae combined with Radix Astragali treatment for CHD showed significant improvements among patients with regard to hemorheology, clinical efficacy, and frequency of angina episodes (Ma et al., 2021). Clinical studies have shown that TJC is remarkably effective for diabetic microvascular and macrovascular complications, such as diabetic peripheral neuropathy (Chen et al., 2015), diabetic nephropathy (Li et al., 2019), and diabetic cerebrovascular disease (An and Xiao, 2018). In our previous clinical study, we showed that TJC exerted therapeutic effects on improving cardiac function and exercise tolerance in T2DM-CHD patients, which implied that it could be considered as a potential Chinese-patent medicine for T2DM-CHD treatment. However, the current clinical data of TJC as complementary therapy in the treatment of T2DM-CHD still lacks high-quality clinical research evidence. Therefore, we designed a randomized, double-blinded, multi-center clinical trial to evaluate the efficacy of TJC in improving cardiac function and exercise tolerance in T2DM-CHD patients.

## Methods/Design

### Study Objectives

The purpose of this pilot study was to evaluate the efficacy of TJC in improving cardiac function and exercise tolerance in T2DM-CHD patients. We hypothesized that, combined with Western conventional medicines, TJC would be more effective than placebo in improving cardiac function and exercise tolerance in T2DM-CHD patients. If the hypothesis is true, this study will provide clinical evidence supporting TJC for the treatment of T2DM-CHD. A total of 360 eligible participants will be enrolled in this study over the course of 2 years.

### Study Design

**FIGURE 1:**
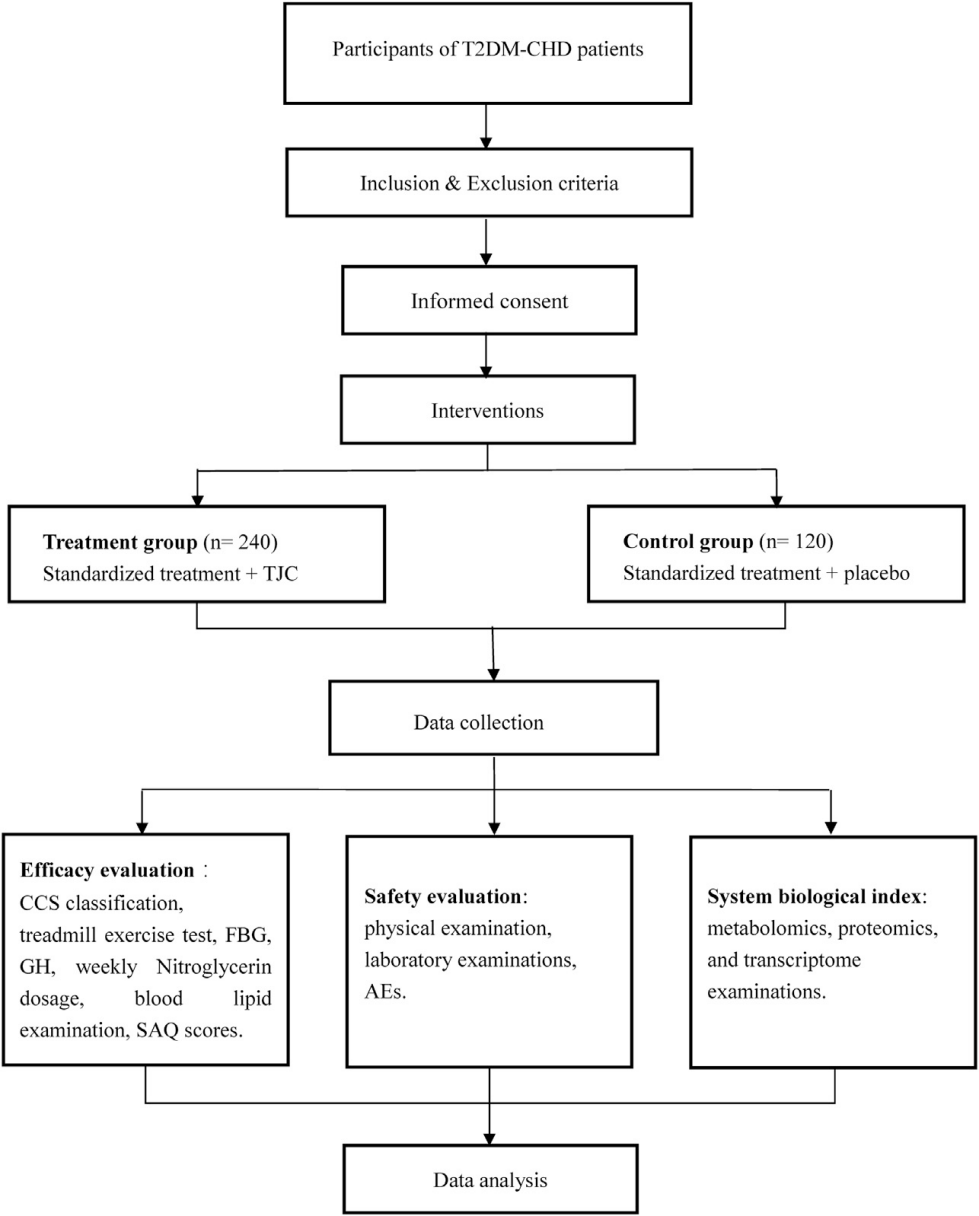
Schedule of the trial. Abbreviations: T2DM- CHD, Type 2 diabetic coronary heart disease; TCJ, Tongmai Jiangtang capsules; CCS, Canadian Cardiovascular Society; FBG, fasting blood glucose; GH, glycosylated hemoglobin; SAQ, Seattle angina questionnaire; AE, adverse event.

This study was designed as a randomized, double-blinded, parallel controlled, multicenter clinical trial. A total of 360 eligible participants will be randomly divided into treatment group and control group in a ratio of 2:1. This study was prospectively registered with Chinese Clinical Trial Registry (ChiCTR2000037491) in September 2020. The study flow is shown in Figure 1.

### Setting and Participants

This study will be conducted at 5 research settings in China (The Affiliated Hospital of Shaanxi University of Chinese Medicine, The Second Affiliated Hospital of Shaanxi University of Chinese Medicine, The Second Affiliated Hospital of Zhejiang Chinese Medical University, Shaanxi Hospital of Traditional Chinese Medicine, and Xi’an Hospital of Traditional Chinese Medicine). Recruitment strategies will include publishing advertisements on local free papers, social media, online publications, and posters displayed in the five participating institutions. Recruitment began in October 2020 and will be completed within 2 years. Patients who consent to participate will be examined and diagnosed by associate chief physicians to confirm their inclusion in the study and will be registered on an online allocation system after written informed consent has been obtained.

### Eligibility Criteria

#### Diagnostic Criteria

Criteria for the diagnosis of T2DM-CHD refer to the Chinese Guidelines for the Prevention and Treatment of Type 2 Diabetes (2017) (Jia et al., 2019) and the Guidelines for the Diagnosis and Treatment of Stable Coronary Heart Disease in 2018 (Fihn et al., 2012). The diagnosis of T2DM generally precedes that of CHD, which appears as a complication of T2DM.

#### Inclusion Criteria

 ◆ Male and female patients aged from 18 to 75 years, inclusively;
 ◆ Patients satisfying the diagnostic criteria of T2DM-CHD, and have a T2DM disease course of more than 6 months;
 ◆ Canadian Cardiovascular Society (CCS) classification ≥ II in the screening phase;
◆ Levels of glycosylated hemoglobin A1c (HbA1c) between 4.0 and 8.0%, inclusively, during the screening period;
◆ Provision of signed informed consent.

#### Exclusion Criteria

 ◆ Type 1 diabetes mellitus, secondary diabetes or failure to specify which type of diabetes;
◆ Patients who have experienced severe hypoglycemic, ketoacidosis, or hyperosmolar coma within 3 months;
◆ Patients experiencing acute coronary syndrome within 3 months;
◆ Evidence of occurrence of cerebrovascular accidents, such as cerebral infarction and cerebral hemorrhage, within 6 months;
◆ History of combined severe heart failure (New York Heart Association class ≥Ⅲ), severe arrhythmia, and other heart diseases;
◆ Patients who have received TCM monotherapy or other TCM prescriptions for the treatment of T2DM and CHD within 14 days;
◆ Allergic constitution or previous allergy to multiple drugs, or known allergy to research drugs and/or its ingredients;
◆ Uncontrolled hypertension (systolic blood pressure [SBP] ≥180 mmHg and/or diastolic blood pressure [DBP] >110 mmHg) or hypotension (SBP <90 mmHg and/or DBP <50 mmHg);
◆ Patients presenting combined severe liver and kidney dysfunction (including patients undergoing dialysis), active liver disease (including primary biliary cirrhosis and unexplained persistent liver dysfunction), or malignant tumors;
◆ Any comorbidities that may affect the evaluation of efficacy and/or safety;
◆ Pregnant or lactating women and those who are planning a pregnancy during the trial or within 3 months after the cessation of the trial;
◆ Participation in other clinical studies within 3 months.

#### Withdrawal Criteria

 ◆ Signs of exacerbation or deterioration clearly related to the intake of the study drug;
◆ Comorbidities, complications, adverse events (AEs), or serious AEs (SAEs) occurring during the study;
◆ Concomitant use of forbidden drugs or receipt of prohibited treatment potentially influencing efficacy and safety;
◆ Subjects who request to withdraw from the study;
◆ Poor compliance by subjects or the amount of drug used does not meet the regulations (less than 80% or more than 120%);
◆ Subjects who have reached the clinical recovery standard before the full course of treatment, and have applied for termination of medication.
◆ Blinding is uncovered or emergency unblinding is required.

### Intervention

This is an add-on study protocol; all the participants will receive standard treatment. The interventions to be used are as follows:

 ◆ Treatment group: TJC, 0.3 g/capsule, op, three capsules/per treatment, three times per day;
◆ Control group: TJC simulant, 0.3 g/capsule, op, three capsules/per treatment, three times per day.

With regard to standard treatment of CHD and T2DM during the intervention period, standard guidelines relative to aspirin, statin lipid-lowering drugs, ACEI or ARB, metformin, sodium-dependent glucose transporters 2 inhibitor, glucagon-like peptide-1 receptor agonists will be used. Substitution with TCM having similar composition and/or efficacy to TJC is not permitted. Researchers should record the concomitant medication truthfully, and maintain the dosage stability during the trial.

### Study Procedure

A time schedule of the study procedures is presented in Table 1. The study will include screening/baseline period and treatment period. The duration of the screening/baseline period will be 2 weeks, while the treatment period will be 3 months. Patients will be requested to provide informed consent in writing before the screening period following a full explanation of the study.

**TABLE 1:** Study schedule.

Research phase	Screening/baseline	Treatment phase		
Number of visits	Visit 1	Visit 2	Visit 3	Visit 4
Visit time point	D-14–0	Week 4 ± 4 days	Week 8 ± 4 days	Week 12 ± 4 days
Data collection				
Written informed consent	●			
Inclusion/exclusion criteria	●			
Demographics	●			
Obtain central random number	●			
Previous history, medical history, and allergies	●			
Comorbidities and co-medications	●			
Access to internet hospital "Dr Tao" platform	●			
Safety evaluation				
Vital signs and physical examination	●	●	●	●
Blood routine	●			●
Urine routine	●			●
Blood biochemistry testing (AST, AKP, TBIL, GGT, Cr, BUN)	●			●
Coagulation testing (PT, APTT, TT, FIB)	●			●
12-Lead ECG	●			●
Urine pregnancy test	●			●
Efficiency evaluation				
CCS Classification	●	●	●	●
Treadmill exercise test	●			●
Fasting blood glucose	●			●
Glycosylated hemoglobin	●			●
Weekly nitroglycerin dosage	●	●	●	●
Blood lipid examination (TC, TG, HDL-C, LDL-C)	●			●
Seattle angina questionnaire scores	●	●	●	●
Other jobs				
Distribute experimental drugs	●	●	●	
Recall test drug		●	●	●
Record AEs and combined medications		●	●	●
Compliance judgment		●	●	●

Abbreviations: ECG, electrocardiogram; CCS, Canadian Cardiovascular Society; ALT, alanine aminotransferase; AST, aspartate aminotransferase, AKP, alkaline phosphatase; TBIL, total bilirubin; GGT, *γγ*-glutamyl transferase; Cr, creatinine; BUN, blood urea nitrogen; PT, prothrombin time; APTT, activated partial thromboplastin time; TT, thrombin time; FIB, fibrinogen; TC, total cholesterol; TG, triglyceride; HDL-C, high-density lipoprotein cholesterol; LDL-C, low-density lipoprotein-cholesterol; AE, adverse event.

### Outcome Measures

#### Primary Outcome

In this study, the primary outcome will be graded according to the CCS classification (Sangareddi et al., 2004). We will compare the difference between the two groups in the proportion of subjects whose CCS classification decreased ≥1 after 12 weeks of medication.

#### Secondary Outcomes

The secondary outcomes of the study medication will be determined by:

 ◆ Treadmill exercise test (conducted partially by subjects). The evaluation indexes include total treadmill exercise time, the appearance of ST-segment 1 mm depression time, the duration of exercise-limited angina, Duke treadmill score (Salokari et al., 2019), rate-pressure product, metabolic equivalents. The amounts of ST-segment deviations is defined as the maximal deviated values (mm) of ST segments on any lead.
◆ Fasting blood glucose and glycosylated hemoglobin (HbA1c);
◆ Weekly nitroglycerin dosage;
◆ Blood lipid examinations including total cholesterol (TC); triglyceride (TG); high-density lipoprotein cholesterol (HDL-C); low-density lipoprotein-cholesterol (LDL-C);
◆ Seattle angina questionnaire (SAQ) scores (Chan et al., 2014);

### Safety Assessment

Safety evaluation indicators include physical examination, vital signs (heart rate, respiration, body temperature, and blood pressure), laboratory examinations, and AEs. Laboratory examinations will include routine blood tests, routine urinalysis, blood biochemistry, coagulation, blood lipids, fasting blood glucose, HbA1c, and 12-lead electrocardiogram. Specifically, blood biochemistry will include alanine aminotransferase (ALT), aspartate aminotransferase (AST), alkaline phosphatase (AKP), total bilirubin (TBIL), γ-glutamyl transferase (GGT), creatinine (Cr), blood urea nitrogen (BUN). Coagulation indices include prothrombin time (PT), activated partial thromboplastin time (APTT), thrombin time (TT), fibrinogen (FIB). Blood lipids examinations will include TC, TG, HDL-C, and LDL-C.

During the treatment period, investigators should pay attention to observe the AEs and unanticipated toxic side effects (including symptoms, signs, and laboratory tests). Regardless of whether the AE is related to the trial drug, it must be recorded in case report form (CRF) in detail, including the occurrence time, symptoms, signs, degree, duration, laboratory test indicators, treatment methods and results, elapsed time, and follow-up time. If instances of an angina attack, subjects should take one nitroglycerin tablet under the tongue at a time, and can repeat one tablet every 5 min until the pain is relieved. If the pain persists after the total amount reaches three tablets within 15 min, medical attention will be immediately requested.

### System Biological Index

Twenty subjects per group will be randomly selected for metabolomics, proteomics, and transcriptome examinations to explore the biomarkers of TJC in treating T2DM-CHD.

Blood collection requirements will consist of fasting for 10 h before sampling at study start (0 weeks) and after treatment (12 weeks), a single blood collection 5 ml, samples will be centrifuged, and serum stored in an EP tube at −70°C; Urine collection requirements will consist of fasting for 10 h before taking medicine (water is permitted), and fasting (both food and water) for 2 h before urine sampling (−1 h) to 2 h after taking the medicine, drinking water quantitatively for 2–8 h after taking the medicine, and eating low-fat meals 4 h after taking the medicine. Urine will be collected and transferred to EP tubes before taking the medicine (0 h) and after treatment (12 weeks), and kept frozen at −70°C.

### Sample Size Calculation

In this study, the sample size is determined by the proportion of subjects whose CCS classification decreased ≥1 after 12 weeks of medication. According to the results of our clinical trials (unpublished data) and our clinical experience, after a 12 weeks intervention, we assume the proportion of subjects whose CCS classification will decrease by ≥ 1 will be 85% in the treatment group and 70% in the placebo control group. Given a type I error rate of *α* = 0.025, a power of 80% (type II error rate of *β* = 0.2), and considering of 20% possible dropout rate, 236 patients should be allocated to the treatment group and 118 patients in the placebo group. For the convenience of randomization, the final sample size was set at 240 cases in the treatment group and 120 cases in the placebo group.

### Randomization

Subjects who sign the informed consent will be randomly assigned a unique screening number through the central randomization system for screening-related examinations and assessments. Eligible subjects will be enrolled according to the time sequence and drug number. The statistical analysis uses the PROC-PLAN process of SAS software to generate computerized random assignment tables. Drugs used in the trial will be coded according to a random allocation table and then randomly assigned to subjects. Note that patients who failed to screen but are suitable for re-screening must use the original screening number; the drug number of the patients who have been screened successfully but have not received the treatment cannot be reassigned to others. The next successfully screened patient will be assigned the next drug number in sequence.

### Blindness

All researchers, subjects, physicians, drug administrators, and dispensing nurses will be blinded to the type of treatment until the study is completed. Blinding is completed by the person in charge of the clinical research unit, the sponsor, and the statisticians. Unblinding is permissible only in the case of the occurrence of a 3-point major adverse cardiovascular event (3-point MACE), including cardiovascular death, non-fatal myocardial infarction, or non-fatal stroke. The patient’s group allocation will be obtained from the drug administrators. The investigator will contact the inspector and report the reasons for unblinding within 24 h. The precise cause of unblinding, the date of AEs, the treatment situation, and the results will be reported in the case report form (CRF) and signed by the administrator.

TJC and placebo are uniformly packaged, and provided by *Baoding Tianhao* Pharmaceutical Co., Ltd. The main components of the placebo will include starch, picric acid, and dextrin, and with its appearance, size, color, dosage form, weight, taste and smell similar to TJC. Furthermore, the number indicated on the test drug packaging will be blinded. The standard label of research drugs will include the drug number, name of the clinical research drug, indications, usage and dosage, course of treatment, storage conditions, batch number, expiration date, and unit providing the test drug.

### Data Collection and Management

The investigator will input the original data into the CRF accurately in strict accordance with the trial protocol and in a timely fashion based on the original observations of participants. To ensure the accuracy of the data, two personnel specializing in data entry should undertake double-entry and proofreading independently. The auditor shall monitor whether all CRFs have been completed and are consistent with the original data, and issue questions at any time in case of any problems. If errors and omissions are made, the researcher shall be corrected promptly.

### Monitoring

To further ensure the quality of clinical trials, the basic principles of "quality by design" and in-process control will be followed (Qu et al., 2019). Before the start of the study, the sponsor will conduct training for the researchers and physicians involved in all centers to ensure the uniformity of administration of medications. The sponsor entrusts the inspector to carry out systematic inspection on the clinical trial and to inspect the trial according to the Good Clinical Practice (GCP) principles to ensure that the test scheme is carried out following the provisions and that the data recorded in the case record form are the same as the original data.

### Statistical Analysis

The statistical analysis plan will be specified before the final data analyses. The statistical analyses will be undertaken via Statistical Analysis System 9.4 (SAS Institute, Inc. Cary, NC) by the Department of Medical Statistics, at the National Center for Cardiovascular Diseases, Beijing. Data from all patients who undergo randomization will analyzed according to the intention-to-treat (ITT) principle. Consistent with the CONSORT statement and ITT principle, the last observation carried forward method will be used for missing values.

For quantitative data, we will calculate the mean, standard deviation, median, minimum, maximum, and interquartile range. For qualitative data, we will describe various frequencies or percentages. The student’s t-test will be used for quantitative data with normal distribution, while the Wilcoxon rank sum test will be used for quantitative data without normal distribution. The chi-square test or Fisher’s exact test will be used for qualitative data. We will analyze intra-group or inter-group differences before and after treatment. Concerning primary outcome, the effectiveness rate of patients whose CCS classification decreased ≥1 after 12 weeks of medication, the differences intra-group or inter-group will be compared using the CMH chi-square test adjusted for clinical site. We will include sex, center, baseline CCS classification, baseline HbA1c level, and baseline lipid levels as covariates in an analysis of covariance (ANCOVA) for statistical differences between groups by reducing the error variance. Statistical Significance will be assumed at a two-sided p-value less than 5%. The relative risk with corresponding 95% confidence interval (CI) to compare dichotomous variables will be calculated.

### Current Status

Patient recruitment for the trial began in October 2020. The relevant protocol was registered at http://www.chictr.org.cn on August 28, 2020. The trial registration number is ChiCTR2000037491.

## Discussion

The diabetic environment promotes the development of CHD through multiple mechanisms, which include glycolipid metabolic disorders, oxidative stress, and increased inflammatory cytokine production (Yahagi et al., 2017). As a Chinese patent medicine, TCJ has been widely used in China as a component of T2DM treatment. Clinical studies have shown that TJC has a significant effect on glycolipid metabolism and diabetes vascular-related complications. Clinical findings have also demonstrated that fasting blood glucose (FPG), 2 h postprandial blood glucose (2-h PBG), HbA1c, TG, TC, and LDL-C levels in TJC group were significantly lower than those of placebo group (Eremire et al., 2019). Moreover, TJC can enhance the immune function and reduce the inflammatory response as shown by analyzing the immune inflammation indicators and had no obvious toxic and side effects on T2DM patients (Eremire et al., 2019). Yang et al. 2016 established a diabetic STZ-induced rat model to investigate the effects of TJC on diabetic neurovascular complications and confirmed that TCJ could improve the footprint gait parameters, nerve conduction velocity, and peripheral nerve pathological injury (Yang et al., 2016).

The representative active ingredients in TJC include calycosin7-O-β-D-glucopyranoside, ononin, formononetin, heterophyllin B, and gypenoside (Yang and Wang, 2020). Experimental studies have demonstrated that calycosin7-O-β-D-glucopyranoside, ononin, formononetin, and gypenoside can significantly decrease blood glucose and lipid levels, which contribute to release lipid metabolism disorder and can effectively remove reactive oxygen species and alleviate oxidative damage (Müller et al., 2012; Sham et al., 2017; Liu et al., 2019). As an effective component of TJC, formononetin can significantly reduce the expression of inflammatory factors such as tumor necrosis factor-α and interleukin-1β (Gu et al., 2014). Heterophyllin B inhibited the lipopolysaccharide-induced inflammation and apoptosis through the phosphoinositide 3-kinase/protein kinase B signaling pathways and has been proposed as a potential therapeutic target for the treatment of inflammatory diseases (Yang et al., 2018). Gypenosides can improve diabetic cardiomyopathy and CHD by inhibiting reactive oxygen species-mediated NLRP3 inflammasome activation (Lee et al., 2013; Zhang et al., 2018b). Thus, cumulative studies to date have evaluated the benefits of TJC in improving glycolipid metabolism, oxidative stress, and inflammatory responses. In addition, our previous studies have shown TJC can uniquely improving the exercise tolerance in T2DM-CHD. However, there have been no relevant randomized controlled trials conducted to date, and we believe that our study will contribute to a better understanding on the efficacy of TJC in T2DM-CHD.

Several limitations need to be considered, however. First, this study will not recorded intervene on patients' diet and exercise, which may have an impact on the level of glucose and lipid, and cardiovascular events (Ades, 2001). Second, AEs will only be recorded and processed during the 3 months intervention period, which is a relatively short period, but the short-term results could encourage further prospective studies with different treatment regimens and longer follow-up. Finally, even in multicenter trials, measurement errors from laboratory testing and individual differences in the process of CCS classification are inevitable, which may possibly lead to different efficacies of TJC. More effort should be made to optimize the deficiencies and answer these questions in future studies.

## Conclusion

This study will investigate the therapeutic potential of TJC as a combination drug. The results of the study will provide reliable clinical research evidence for the application of TJC in improving cardiac function and exercise tolerance in T2DM-CHD patients.

## Data Availability

The original contributions presented in the study are included in the article/Supplementary Material, further inquiries can be directed to the corresponding authors. After the completion of the study, the data and results of the trial will be disseminated to the general public through scientific conferences, presentations, and open-access medical journals.
